# *catena*-Poly[[bis­[(dicyanamido)silver(I)](*Ag*—*Ag*)]-μ_2_-4,4′-bipyridine-κ^2^ *N*:*N*′]

**DOI:** 10.1107/S1600536809050491

**Published:** 2009-11-28

**Authors:** Jinfang Zhang

**Affiliations:** a Molecular Materials Research Center, Scientific Research Academy, School of Chemistry and Chemical Engineering, Jiangsu University, Zhenjiang 212013, People’s Republic of China

## Abstract

In the title compound, [Ag_2_(C_2_N_3_)_2_(C_10_H_8_N_2_)]_*n*_, the Ag atoms, lying on inversion centers, are separated by 3.3226 (12) Å. Each Ag atom is connected by one bridging 4,4′-bipyridine [Ag—N = 2.177 (4)Å] and a terminal dicyanamide [Ag—N = 2.108 (4) Å]. The Ag—Ag interactions play a key role in constructing a unique neutral polymeric chain.

## Related literature

For the designed syntheses of metal-organic compounds, see: Eddaoudi *et al.* (2001 ▶); Zhang *et al.* (2008 ▶, 2009*a* ▶,*b* ▶). For their applications, see: Banerjee *et al.* (2008 ▶); Zhang *et al.* (2007 ▶).
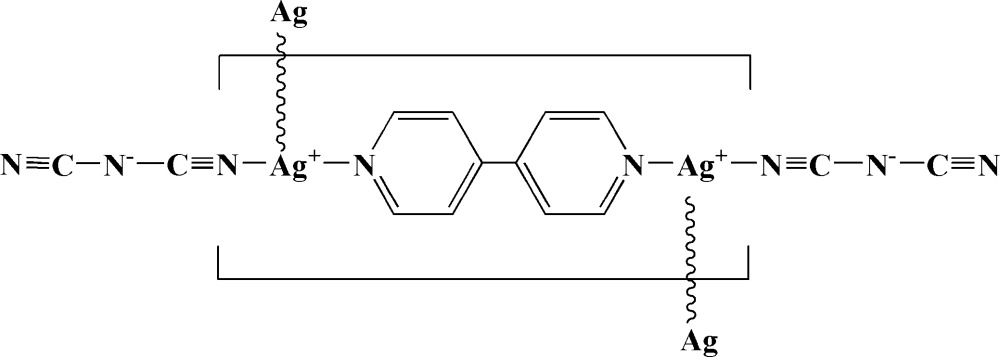


## Experimental

### 

#### Crystal data

[Ag_2_(C_2_N_3_)_2_(C_10_H_8_N_2_)]
*M* *_r_* = 252.01
Triclinic, 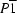

*a* = 6.1867 (12) Å
*b* = 7.8344 (16) Å
*c* = 7.9649 (16) Å
α = 88.83 (3)°
β = 84.09 (3)°
γ = 77.54 (3)°
*V* = 374.95 (13) Å^3^
*Z* = 2
Mo *K*α radiation
μ = 2.63 mm^−1^
*T* = 293 K
0.2 × 0.16 × 0.12 mm

#### Data collection

Rigaku Saturn724+ diffractometer
Absorption correction: multi-scan (*SADABS*; Sheldrick, 1996 ▶) *T*_min_ = 0.407, *T*_max_ = 0.664
2474 measured reflections
1358 independent reflections
1315 reflections with *I* > 2σ(*I*)
*R*_int_ = 0.022

#### Refinement

*R*[*F* ^2^ > 2σ(*F* ^2^)] = 0.033
*wR*(*F* ^2^) = 0.103
*S* = 1.24
1358 reflections
110 parameters
H-atom parameters constrained
Δρ_max_ = 0.67 e Å^−3^
Δρ_min_ = −0.70 e Å^−3^

### 

Data collection: *CrystalClear* (Rigaku, 2008 ▶); cell refinement: *CrystalClear*; data reduction: *CrystalClear*; program(s) used to solve structure: *SHELXS97* (Sheldrick, 2008 ▶); program(s) used to refine structure: *SHELXL97* (Sheldrick, 2008 ▶); molecular graphics: *SHELXTL* (Sheldrick, 2008 ▶); software used to prepare material for publication: *SHELXTL*.

## Supplementary Material

Crystal structure: contains datablocks I, global. DOI: 10.1107/S1600536809050491/pv2240sup1.cif

Structure factors: contains datablocks I. DOI: 10.1107/S1600536809050491/pv2240Isup2.hkl

Additional supplementary materials:  crystallographic information; 3D view; checkCIF report

## Figures and Tables

**Fig. 1.**
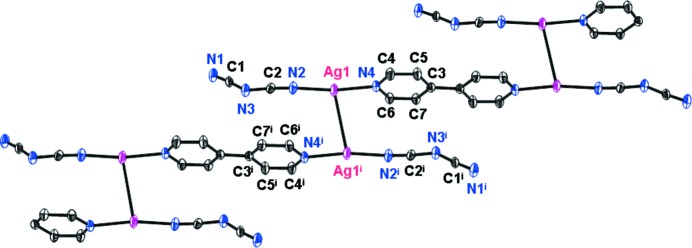
The molecular structure of the title compound, with atom labels and 30% probability displacement ellipsoids; H atoms have been omitted for clarity. Symmetry code: i = -x+2, -y+1, -z+1.

## supplementary crystallographic information

### Comment

The designed syntheses of metal-organic compounds have attracted great attention in recent years because of not only their intriguing structures (Eddaoudi *et al.*, 2001; Zhang *et al.*, 2008) but also their potential applications.(Banerjee *et al.*, 2008; Zhang *et al.*, 2007). The flexible and rigid bridging ligands can play different roles in constructing metal-organic frameworks. The tilte compound, (I), was constructed by employing a flexible, dicyanamide, and a rigid, 4,4'-bipyridine ligand through diffusion reactions. In this paper, the crystal structure of (I) is presented.

As illustrated in Fig. 1, 4,4'-bipyridine acts as a bridgeing ligand to connect two Ag atoms. Dicyanamide usually acts as a briding ligand to construct metal-organic compounds (Zhang *et al.*, 2009*a,b*). However, in the tilte compound, it is linked to only one Ag atom. Ag—Ag bonds [3.3226 (12) Å] play a key role in constructing a unique one-dimensional neutral chain.

### Experimental

Ag(NO_3_) (68.0 mg, 0.4 mmol) and NaN(CN)_2_ (178.2 mg, 2 mmol) were added into 3 ml dimethylformamide with thorough stirring for 5 minutes. After filtration, the colorless filtrate was carefully laid on the surface of a solution of 4,4'-bipyridine (78.0 mg, 0.5 mmol) in 8 ml *i*-PrOH. Colorless prismatic crystals were obtained after five days.

### Refinement

H atoms were positioned geometrically and refined with riding model, with *U*_iso_ = 1.2*U*_eq_ for pyridyl H atoms, the C—H bonds are 0.93 Å in pyridyl.

### Crystal data

**Table:**

[Ag_2_(C_2_N_3_)_2_(C_10_H_8_N_2_)]	*Z* = 2
*M**_r_* = 252.01	*F*(000) = 242
Triclinic, *P*1	*D*_x_ = 2.232 Mg m^−^^3^
Hall symbol: -P 1	Mo *K*α radiation, λ = 0.71073 Å
*a* = 6.1867 (12) Å	Cell parameters from 1687 reflections
*b* = 7.8344 (16) Å	θ = 2.6–28.7°
*c* = 7.9649 (16) Å	µ = 2.63 mm^−^^1^
α = 88.83 (3)°	*T* = 293 K
β = 84.09 (3)°	Prism, colorless
γ = 77.54 (3)°	0.2 × 0.16 × 0.12 mm
*V* = 374.95 (13) Å^3^	

### Data collection

**Table:**

Rigaku Saturn724+ diffractometer	1358 independent reflections
Radiation source: fine-focus sealed tube	1315 reflections with *I* > 2σ(*I*)
graphite	*R*_int_ = 0.022
dtprofit.ref scans	θ_max_ = 25.5°, θ_min_ = 2.6°
Absorption correction: multi-scan (*SADABS*; Sheldrick, 1996)	*h* = −7→7
*T*_min_ = 0.407, *T*_max_ = 0.664	*k* = −6→9
2474 measured reflections	*l* = −9→9

### Refinement

**Table:**

Refinement on *F*^2^	Secondary atom site location: difference Fourier map
Least-squares matrix: full	Hydrogen site location: inferred from neighbouring sites
*R*[*F*^2^ > 2σ(*F*^2^)] = 0.033	H-atom parameters constrained
*wR*(*F*^2^) = 0.103	*w* = 1/[σ^2^(*F*_o_^2^) + (0.0601*P*)^2^ + 0.1547*P*] where *P* = (*F*_o_^2^ + 2*F*_c_^2^)/3
*S* = 1.24	(Δ/σ)_max_ < 0.001
1358 reflections	Δρ_max_ = 0.67 e Å^−^^3^
110 parameters	Δρ_min_ = −0.70 e Å^−^^3^
0 restraints	Extinction correction: *SHELXL97* (Sheldrick, 2008), Fc^*^=kFc[1+0.001xFc^2^λ^3^/sin(2θ)]^-1/4^
Primary atom site location: structure-invariant direct methods	Extinction coefficient: 0.055 (7)

### Special details

**Table:**

Geometry. All e.s.d.'s (except the e.s.d. in the dihedral angle between two l.s. planes) are estimated using the full covariance matrix. The cell e.s.d.'s are taken into account individually in the estimation of e.s.d.'s in distances, angles and torsion angles; correlations between e.s.d.'s in cell parameters are only used when they are defined by crystal symmetry. An approximate (isotropic) treatment of cell e.s.d.'s is used for estimating e.s.d.'s involving l.s. planes.
Refinement. Refinement of *F*^2^ against ALL reflections. The weighted *R*-factor *wR* and goodness of fit *S* are based on *F*^2^, conventional *R*-factors *R* are based on *F*, with *F* set to zero for negative *F*^2^. The threshold expression of *F*^2^ > σ(*F*^2^) is used only for calculating *R*-factors(gt) *etc*. and is not relevant to the choice of reflections for refinement. *R*-factors based on *F*^2^ are statistically about twice as large as those based on *F*, and *R*- factors based on ALL data will be even larger.

### Fractional atomic coordinates and isotropic or equivalent isotropic displacement parameters (Å^2^)

**Table:**

	*x*	*y*	*z*	*U*_iso_*/*U*_eq_	
Ag1	0.83031 (5)	0.39268 (4)	0.61182 (3)	0.0570 (3)	
N1	0.6869 (7)	0.6445 (6)	0.3950 (4)	0.0626 (10)	
N2	0.7664 (7)	0.5131 (6)	−0.1486 (5)	0.0544 (9)	
N3	0.6751 (7)	0.7081 (5)	0.0944 (5)	0.0518 (9)	
N4	0.8957 (6)	0.2168 (4)	0.3957 (4)	0.0432 (8)	
C1	0.6855 (6)	0.6651 (5)	0.2524 (5)	0.0428 (8)	
C2	0.7278 (6)	0.5953 (5)	−0.0277 (5)	0.0416 (8)	
C3	0.9785 (6)	0.0426 (5)	0.0830 (5)	0.0367 (8)	
C4	0.7354 (7)	0.2209 (6)	0.2947 (6)	0.0521 (10)	
H4	0.5933	0.2842	0.3301	0.063*	
C5	0.7682 (7)	0.1371 (6)	0.1417 (6)	0.0511 (10)	
H5	0.6497	0.1434	0.0772	0.061*	
C6	1.0962 (7)	0.1231 (5)	0.3420 (5)	0.0447 (9)	
H6	1.2110	0.1175	0.4100	0.054*	
C7	1.1423 (6)	0.0341 (5)	0.1914 (5)	0.0431 (8)	
H7	1.2844	−0.0324	0.1617	0.052*	

### Atomic displacement parameters (Å^2^)

**Table:**

	*U*^11^	*U*^22^	*U*^33^	*U*^12^	*U*^13^	*U*^23^
Ag1	0.0661 (4)	0.0685 (3)	0.0347 (3)	−0.0119 (2)	0.00158 (18)	−0.02256 (18)
N1	0.084 (3)	0.067 (2)	0.033 (2)	−0.008 (2)	−0.0024 (19)	−0.0116 (16)
N2	0.064 (2)	0.064 (2)	0.0322 (19)	−0.0080 (18)	−0.0025 (16)	−0.0155 (16)
N3	0.067 (2)	0.0520 (19)	0.0328 (19)	−0.0039 (16)	−0.0054 (16)	−0.0089 (14)
N4	0.0522 (19)	0.0447 (17)	0.0315 (17)	−0.0072 (14)	−0.0042 (14)	−0.0108 (13)
C1	0.046 (2)	0.0467 (19)	0.035 (2)	−0.0082 (15)	−0.0020 (16)	−0.0148 (15)
C2	0.0421 (19)	0.051 (2)	0.0298 (19)	−0.0065 (15)	−0.0029 (15)	−0.0036 (16)
C3	0.0445 (19)	0.0327 (16)	0.0321 (19)	−0.0068 (14)	−0.0026 (15)	−0.0023 (14)
C4	0.043 (2)	0.060 (2)	0.047 (2)	0.0017 (17)	−0.0021 (18)	−0.0214 (19)
C5	0.043 (2)	0.061 (2)	0.045 (2)	0.0015 (17)	−0.0080 (17)	−0.0224 (19)
C6	0.049 (2)	0.049 (2)	0.035 (2)	−0.0064 (16)	−0.0091 (17)	−0.0074 (16)
C7	0.0411 (19)	0.046 (2)	0.038 (2)	0.0005 (15)	−0.0065 (16)	−0.0082 (15)

### Geometric parameters (Å, °)

**Table:**

Ag1—N2^i^	2.108 (4)	N4—C4	1.334 (6)
Ag1—N4	2.177 (4)	C3—C7	1.387 (6)
Ag1—N1	2.661 (4)	C3—C5	1.390 (6)
Ag1—Ag1^ii^	3.3226 (12)	C3—C3^iv^	1.467 (8)
N1—C1	1.144 (5)	C4—C5	1.372 (6)
N2—C2	1.145 (6)	C4—H4	0.9300
N2—Ag1^iii^	2.108 (4)	C5—H5	0.9300
N3—C2	1.296 (6)	C6—C7	1.373 (6)
N3—C1	1.301 (6)	C6—H6	0.9300
N4—C6	1.331 (5)	C7—H7	0.9300
			
N2^i^—Ag1—N4	167.60 (15)	C7—C3—C3^iv^	122.8 (4)
N2^i^—Ag1—N1	105.43 (14)	C5—C3—C3^iv^	121.4 (4)
N4—Ag1—N1	86.07 (12)	N4—C4—C5	123.8 (4)
N2^i^—Ag1—Ag1^ii^	105.25 (12)	N4—C4—H4	118.1
N4—Ag1—Ag1^ii^	84.71 (10)	C5—C4—H4	118.1
N1—Ag1—Ag1^ii^	57.43 (10)	C4—C5—C3	120.0 (4)
C1—N1—Ag1	139.3 (4)	C4—C5—H5	120.0
C2—N2—Ag1^iii^	172.6 (4)	C3—C5—H5	120.0
C2—N3—C1	123.1 (4)	N4—C6—C7	123.4 (4)
C6—N4—C4	116.4 (4)	N4—C6—H6	118.3
C6—N4—Ag1	123.9 (3)	C7—C6—H6	118.3
C4—N4—Ag1	118.8 (3)	C6—C7—C3	120.5 (4)
N1—C1—N3	173.2 (5)	C6—C7—H7	119.7
N2—C2—N3	171.4 (4)	C3—C7—H7	119.7
C7—C3—C5	115.8 (4)		

Symmetry codes: (i) *x*, *y*, *z*+1; (ii) −*x*+2, −*y*+1, −*z*+1; (iii) *x*, *y*, *z*−1; (iv) −*x*+2, −*y*, −*z*.
